# Post-stroke cognitive impairment at 3 months

**DOI:** 10.4103/0972-2327.61276

**Published:** 2010

**Authors:** Uma Sundar, Sikandar Adwani

**Affiliations:** Neurology services, Internal Medicine, Lokmanya Tilak Municipal Medical College and Hospital, Sion, Mumbai, India

**Keywords:** Stroke, cognition, executive dysfunction

## Abstract

**Background:**

Vascular cognitive impairment, being the only treatable cause of dementia in the early stages, and having a diverse etiology, requires sensitive criteria for definition.

**Aim:**

This study aimed to study cognitive functions at 3 months post-stroke, utilising the Mini mental scale examination (MMSE) and the Frontal assessment battery (FAB), and to correlate the same with subgroups of ischemic stroke.

**Results:**

164 patients were studied, 108 of these having multiple infarcts. Pure cortical infarcts were seen in 41 patients. At 3 months, 112/164 patients had MMSE more than 24, with no frontal executive dysfunction. MMSE score less than 24 was seen in 24 patients, all of them having FAB score below 10. Normal MMSE with impaired FAB was seen in 28 patients.

**Conclusions:**

Impairment on either the MMSE or the FAB was thus seen in 31.7% patients (52/164), at 3 months after stroke. FAB impairment alone, with MMSE in normal range, was seen in 28/52 (53.8%) patients. Memory was significantly more commonly affected in muti-infarct strokes as compared to single infarcts. Frontal executive dysfunction was not significantly different when single and multiple infarcts, or cortical and subcortical infarcts, were compared.

## Introduction

Vascular cognitive impairment (VCI) has a varied and diverse etiology; the various forms include cognitive impairment following single strategic infarcts, subcortical VCI, and multi-infarct dementia.[1] VCI, rather than vascular dementia (Va D), is the more appropriate term, as the correct objective should be to identify the condition before it develops into frank dementia. This is particularly important as, apart from age, ‘vascular risk factors’ are the most important and presently the only treatable precursor to dementia. [2–4] However, the recognition and definition of VCI is fraught with difficulties. No uniformly applicable criteria have been laid down; in fact, the criteria most often applied suffer from a number of drawbacks.[5,6] The NINDS-AIREN/California criteria stress ‘memory impairment’ as a mandatory criterion.[7,8] This is more suitable for the diagnosis of Alzheimer's disease (AD), and may not be true for subcortical vascular ischemia (SCVI).[9] The ischemic scale, which separates Va D from other causes based on vascular risk factors and previous strokes, may no longer be as discriminatory as previously thought, with the dawning understanding that many of the vascular risk factors are shared between AD and Va D; furthermore, degenerative and vascular conditions may supplement and augment each other.[10,11]

The ICD 10 places emphasis on ‘patchy cognitive deficits’ which, again, is more suited for the diagnosis of AD; it is inapplicable in the case of subcortical dementia due to leukoaraiosis/multiple lacunar infarcts, where the dementia may be slowly progressive, may affect only the frontal executive functions, and may not be related to any clinical index stroke.[12] Hence, collection of data pertaining to cognitive abilities post stroke (at various periods from 3 months to several years), without the application of any set criteria, is needed for a better understanding of this entity.

### Aims of the study

The aim of this study was to assess cognitive dysfunction at 3 months after ischemic stroke. Additionally, the aim was also to assess frontal executive functions over and above that assessed by the routine mini mental scale examination (MMSE), as well as to evaluate the degree and type of cognitive dysfunction in different subgroups of ischemic stroke (viz multiple*vs* single infarcts and cortical*vs* subcortical strokes).

## Materials and Methods

Serial recruitment of 164 patients of new stroke was done over a 1.5-year period (2004–2006) from the medical wards of a tertiary care public hospital. The ages of the subjects ranged from 52 to 81 years (mean 63 years). There were 103 male and 61 female patients. Only literate patients were included; 93% of the subjects had studied up to the 5^th^ grade at least. The patients’ clinical and imaging data at presentation were noted. They were followed up and, at 3 months post stroke, the modified Folstein's mini mental scale examination (MMSE – Hindi version) and frontal assessment battery (FAB) were performed on them.[13,14] Patients were excluded from the study if there was a history suggestive of prior clinical stroke or cognitive dysfunction. The presence of depression (as per the DSM-4 criteria) at 3 months post stroke also resulted in exclusion from the study. Data were compared between single and multiple infarcts and between cortical and subcortical infarcts.

SPSS for Windows, version 11, was used for statistical analysis.

## Results

The 164 patients recruited included 56 patients with single infarcts and 108 with multiple infarcts. Of the 56 single infarcts, 28 were pure cortical infarcts. The 108 multiple infarcts included 13 pure cortical, 32 subcortical, and 63 mixed infarcts. Pure cortical infarcts (single or multiple) totaled 41, pure subcortical infarcts numbered 60 (single or multiple), and mixed cortical and subcortical infarcts numbered 63. The subcortical-mixed group (single or multiple) totaled 123.

At 3 months’ cognition testing, 112 patients had an MMSE score more than or equal to 24; they did not have any frontal executive dysfunction (FED) [Table 1]. In these 112 patients, the mean MMSE score lost was 4. The modalities that were most commonly lost were 3-stage command (83/112), writing (89/112), attention and concentration (72/112), and recall (63/112) [Table 2].

**Table 1:** MMSE scores and frontal executive dysfunction

MMSE score	No. of patients with normal MMSE and FED* (28)	No. of patients with normal MMSE and no FED (112)
28–30	15	32
26, 27	9	42
24, 25	4	38

* FED-Frontal executive dysfunction. Scores were uniformly less than 15/18.

**Table 2:** MMSE modalities lost in patients with normal MMSE scores

MMSE modality lost	Patients with FED* (28)	Patients with no FED (112)
Attention and concentration	14	72
Recall	9	63
Praxis	14	13
Orientation in place	13	52
Orientation in time	19	49
Naming	12	21
Repetition	10	41
3-stage command	13	83
Writing	21	89

* FED: Frontal executive dysfunction.

All patients (24) with MMSE less than 24 had impaired frontal executive functions, the FAB being uniformly less than 10. In 13 of these 24 patients, it was not possible to perform the complete frontal battery due to markedly impaired attention and concentration.

The group having normal MMSE but abnormal frontal executive functions comprised 28 patients. As seen inTable 1, 24/28 of these patients had MMSE scores that were in the highest range, i.e., 27–30. The commonest modalities in MMSE lost in this group were writing (21/28), orientation in time (19/28), attention and concentration (14/28), and praxis (14/28) [Table 2]. The scores on the FAB were as follows: 14 patients had FAB scores of 13–15, 13 patients had scores of 10–12, and 1 patient had a score of <9.

Thus, a total of 52 patients were considered to have cognitive impairment in this study: 24 with impaired MMSE score and an additional 28 with impairment on the FAB. The demographic data pertaining to the prevalence of vascular risk factors in the patients studied is shown inTable 3. However, correlation of post-stroke cognitive impairment with vascular risk factors was not statistically possible in this study due to paucity of numbers.

**Table 3:** Epidemiological factors and cognitive impairment following stroke

Epidemiological parameter	Total no.	No. of patients cognitively impaired*
Age 60–70 years	92	27
70–80 years	45	18
>80 years	27	7
Hypertension	84	28
Diabetes mellitus	41	38
Smoking	76	22
Hyperlipidemia	55	14
Education up to 10th	101	32

* Cognitively impaired: MMSE score < 24 or impaired frontal assessment battery. FAB scores were uniformly less than 15/18.

Among those with multiple infarcts, 16/108 (14.81%) had MMSE scores less than 24 plus an FAB score less than 15; an additional 16 (14.81%) patients had impairment on FAB only, while having a normal MMSE score. The FAB impairments included affection of motor series (16 patients), conflicting instructions (10), inhibitory control (8), lexical fluency (9), conceptualization (6), and prehensile behavior (3). The range of the FAB scores in these patients was 7–14, the median score being 10.Table 4 shows the number of impaired patients in the subgroups of multiple cortical infarcts, multiple subcortical infarcts, and multiple mixed infarcts.

**Table 4:** Cognitive dysfunction in multiple infarcts

Cognitive dysfunction	Multiple cortical (13) No. (%)	Multiple subcortical (32) No. (%)	Mixed (63) No. (%)	Total (108) No. (%)
MMSE + FAB	6 (46.15)	5 (15.63)	5 (7.93)	16 (14.81)
FAB only	3 (23.08)	7 (21.86)	6 (9.52)	16 (14.81)
Total	9 (69.23)	12 (37.49)	11 (17.45)	32 (29.63)

Among those with single infarcts, 8/56 (14.2%) had MMSE scores less than 24 plus FAB scores less than 15. An additional 12/56 (21.3%) patients had impaired FAB score only; these included motor series (8 patients), conflicting instructions (7), inhibitory control (6), lexical fluency (7), conceptualization (6), and prehensile behavior (4). The range of the FAB scores in these patients was 11–15, the median score being 13.Table 5 shows details of cognitive impairment in the subgroups of cortical and subcortical single infarcts.

**Table 5:** Cognitive dysfunction in single infarcts

Cognitive dysfunction	Single cortical (28) No. (%)	Single subcortical (28) No. (%)	Total (56) No. (%)
MMSE + FAB	6 (21.43)	2 (7.14)	8 (14.29)
FAB only	5 (17.86)	7 (25)	(21.43)
Total	11 (39.29)	9 (32.14)	(35.71)

Table 6 shows the details of FAB modalities impaired in the subgroups of pure cortical, pure subcortical, and mixed infarcts.

**Table 6:** Cognitive dysfunction in cortical and subcortical infarcts

Cognitive dysfunction	Cortical infarcts (41) No. (%)	Subcortical infarcts (60) No. (%)	Mixed infarcts (63) No. (%)
Impaired MMSE	12 (29.2)	7 (11.66)	5 (7.93)
FED	8 (19.5)	14 (23.3)	6 (9.53)
Motor series	4 (9.76)	9 (15)	6 (9.53)
Conflicting instructions	6 (14.63)	7 (11.66)	6 (11.32)
Inhibitory control	4 (9.76)	6 (10)	5 (7.94)
Lexical fluency	4 (9.76)	9 (15)	8 (12.7)
Conceptua lization	6 (14.63)	7 (11.66)	8 (12.7)
Prehensile behavior	3 (7.32)	2 (4.88)	2 (4.88)

Among the 41 patients with pure cortical infarcts, 21 (51.21%) had impaired MMSE (score < 24) along with impaired FAB. An additional 8 (19.51%) patients had impaired FAB only; this was as follows: motor series (4 patients), conflicting instructions (6), inhibitory control (4), lexical fluency (4), conceptualization (6), and prehensile behavior (3). The range of the FAB scores in these patients was 10–15, the median score being 13.

Among the 60 patients with pure subcortical infarcts, 7 patients (11.67%) had impaired MMSE scores of less than 24. An additional 14 (23.33%) patients had impaired FAB only; this was as follows: motor series (9 patients), conflicting instructions (7), inhibitory control (6), lexical fluency (9), conceptualization (7), and prehensile behavior (2). The range of the FAB scores in these patients was 8–14, the median score being 11.

In the mixed group (cortical and subcortical), 5/63 (7.9%) patients had impaired MMSE and FAB; an additional 6/63 (9.52%) had impaired FAB only, which was as follows: motor series (6 patients), conflicting instructions (6), inhibitory control (5), lexical fluency (8), conceptualization (8), and prehensile behavior (2).

On statistical analysis there was no significant difference in overall cognitive impairment in the multiple infarct group*vs* the single infarct group (29.62%*vs* 35.71%, respectively).

Memory impairment (recall on the MMSE) was specifically analyzed; 34/108 (31.4%) patients in the multiple infarct group and 8/56 (14.2%) patients in the single infarct group had Impaired memory. This difference was statistically significant (*P*<0.005). FED was seen in 14.8% of multiple infarcts and in 21.3% of single infarcts; this difference was statistically not significant.

Total cognitive impairment in pure cortical and pure subcortical Strokes was 70.73% and 35% respy. This difference was statistically significant (*P*<0.005). Pure cortical strokes had FED in 19.5% patients and pure subcortical strokes had FED in 23.3% patients. These values were not significantly different.

## Discussion

Post-stroke VCI has a varied etiology. Additionally, it is closely related to pre-stroke cognitive status. It is possible that previous subclinical strokes could have led to cognitive dysfunction that was circumvented over time by the development of neural networks; however, when new vascular events occur, these networks are gradually rendered dysfunctional, resulting in deficits that are more devastating than the new, index stroke could cause by itself.[15]

In the present study, post-stroke VCI was assessed at 3 months after the index stroke. The 3-month interval was chosen to allow sufficient time to elapse for acute delirium and medical complications, if any, to subside and for the deficit to stabilize.

Of 164 patients studied, 56 had single infarcts and 108 had multiple infarcts. In this study, the presence of a history of ‘previous clinical stroke’ was an exclusion criterion. The presence of 108 multiple infarcts thus highlighted the prevalence of previous unnoticed/subclinical vascular events in the population. It also supports the theory of the development of ‘neural networks’ that serve to circumvent and overcome the neurological dysfunction caused by vascular insults.

Cognitive dysfunction was seen in 31.7% (52/164) of our patients. Patients with either Frontal executive dysfunction, or abnormal MMSE for age or both, were considered to have cognitive dysfunction. In this study, the emphasis was on documenting the range of cognitive functions post-stroke, rather than attempting to fit the findings into predefined criteria for vascular dementia. This was done so as to detect the true range of cognitive deficits post stroke, and to avoid the pitfall of tautology, viz, if ‘memory deficit’ is one of the criteria to define post-stroke cognitive dysfunction, then all the cases detected would necessarily be ‘memory impaired.’ Similarly, if subtle tests of cognitive dysfunction are not included in the testing battery, these would never be identified.[16,17]

In this study, 112/164 (68.29%) patients were ‘cognitively normal’ at the end of 3 months post stroke, having an MMSE more than 24/30 and no frontal lobe dysfunction. Among these 112 patients, the mean MMSE score ‘lost’ was 4, the range being 0–6. The loss was predominantly due to deficits in calculation, recall, details of orientation, and praxis.

In 28 out of 52 (53.8%) patients, although the MMSE was more than 24, significant impairment was detected on frontal executive testing. The MMSE has been validated in numerous studies and the recommended cutoff of 24 is sensitive to the detection of dementia. However, it is insensitive to the early changes of dementia due, in part, to the heavy weightage given to orientation and memory. It is insensitive to cognitive impairment in cerebrovascular disease, multiple sclerosis, and Parkinson's disease, since the impairment in these conditions often involves executive function and attention.[18,19]

In this study, the singularity or multiplicity of infarcts did not appear to influence cognitive dysfunction at 3 months. Hence, the location of infarcts is at least as important as, if not more important than, the number of infarcts. Single strategic infarcts, such as in the dominant anterior or posterior cerebral artery territory, a thalamic infarct, Wernicke's area affection due to involvement of the upper division of the middle cerebral artery, etc., are well known to present as pure cognitive dysfunction.[20,21]

The significantly higher percentage of memory-affected patients in the multiple infarcts group as compared to that in the single infarcts group is not surprising. This difference highlights the role of complex neural networks in sustaining memory.[22,23] In fact, in these patients, the earlier lesions were probably subclinical, as our inclusion criteria specified that the study subjects should not have history of ‘prior clinical strokes.’ The initial ischemic damage may not have left any detectable deficit due to circumvention by neural networks, allowing gradual ‘learning and adaptation.’ However, lesions that developed later in the disease process uncovered previous damage and added new deficits, one of the most prominent being in the area of memory.

In this study, FED was seen in both multiple as well as single infarcts in equal measure. The same was also true of cortical and subcortical infarcts. In previous studies, Vataja*et al* and Godefroy*et al* have shown that infarcts and white matter lesions in the frontal-subcortical circuits may lead to executive dysfunction post-stroke.[24,25]

Frontal executive function comprises the ability to maintain attention and concentration, plan and execute a task, exercise mental flexibility and judgment, generate ideas and words, and interpret abstract thought.[26] The frontal-subcortical circuits and their distinct neurological correlates are well recognized. Disruption of the dorsolateral prefrontal circuit results in poor organizational and set-shifting strategies, and stimulus- bound behavior. Disruption of the orbitofrontal circuit leads to emotional incontinence, disinhibition, obsessive compulsive disorders, and mood disorders, whereas anterior cingulate circuit lesions cause apathy, impaired motivation, and akinetic mutism.[27] Executive dysfunction may be seen in ‘cognitively normal’ elderly patients, in association with subcortical lacunes.[28] As shown by Tullberg*et al*., subcortical ischemic vascular disease preferentially affects frontal lobe functions, regardless of the location of white matter involvement.[29] In the present study, executive functions were assessed only after depression had been ruled out. However, a close correlation between post-stroke FED and depression has been shown by Pohjasvaara*et al*. and Vataja*et al*. In their series, 40.6% patients had post-stroke executive dysfunction, this dysfunction showing association with older age, concomitant depression, lower education level, and with lesions in the anterior circulation.[30,31]

In conclusion, 31.7% patients had cognitive impairment at 3 months post ischemic stroke, either on the MMSE or the FAB. Of the study subjects, 17.07% were impaired on frontal executive functions only. Single and multiple infarcts were not significantly different with respect to post-stroke cognitive impairment, the main difference being that memory affection was significantly commoner in the latter. The importance of the need to be aware of disorders of these specialized functions, which directly impact the quality of life post stroke, cannot be over-emphasized.
